# Allegheny County Medical Society

**Published:** 1890-08

**Authors:** 


### ALLEGHENY COUNTY MEDICAL SOCIETY.

SPECIAL MEETING, June 17th, 1890.

J. A. Lippincott, M. D., President *pro tem.*, in the chair.

### CASE OF SUSPECTED VOLVULUS.

Dr. Batten reported: On Sunday, June 8, I saw a man who had been suffering for several days with intense abdominal pains. There was sickness at the stomach. I gave him a quarter of a grain of morphia sulphate and extract of belladonna every hour. That did not control the pain, and Dr. Pollock was called in consultation. We continued with the belladonna and the sulphate of morphia every hour, and a hypodermic injection of morphia every six hours. This quieted him. The morphia and belladonna were continued steadily, and when the pains became unbearable morphia was injected. He continued in that way, with vomiting, until Friday. I omitted to say we injected warm water into the bowels. On Friday afternoon there was a passage of the bowels, and we omitted the hypodermic injection and the morphia and belladona, and gave him Rochelle salts in small doses. Yesterday he had three hours' sleep. Last night he slept all night. To-day he is well.

Dr. Buchanan: I would like to know if any tumor could be felt.

Dr. Batten: No, but the abdomen was very hard.

Dr. Lange: I would submit that Dr. Batten has given no evidence of intussusception; it might have been a case of ileus, a case of fecal obstruction, or of typhlitis; Dr. Batten has given no evidence of volvulus.

Dr. Batten: It was an obstruction of the bowels. No hard matter came away after the bowels commenced to move.

Dr. Huselton: I would be disposed to criticize the treatment.

I cannot readily understand why he should resort to purgatives in such a case. Of course the opiates would be all right to allay pain and quiet the bowels. It seems to me that purgatives would hardly be proper in such cases.

### CASE OF PUERPERAL CONVULSIONS.

Dr. Duff: About one year ago, I reported a case of puerperal convulsions to this society. I was called to see a young woman of 20, about two weeks before the time of labor. I found general anasarca as well as urine loaded with albumen, and I treated her, as I indicated in my report, by giving her digitalis. About six days after, I was called to see her. She was taken in convulsions, which could only be controlled by chloroform. These we controlled for about twenty-four hours, when they returned. I then dilated and delivered her of a male child. The child lived about a week. The mother made a good recovery and did not have convulsions after delivery. Against my orders, she cohabited with her husband and became pregnant within three months. Before pregnancy her urine was normal. At four months I examined her and found considerable albumen. I then put her upon nitro-glycerine, one drop doses three times a day, with the result that the albuminuria disappeared. At six months the albumen returned, as well as the dropsical condition. She was again put on nitro-glycerine, and there remained a slight trace of albumen during her whole pregnancy. She was confined last Tuesday morning; I applied the forceps, delivered her, and she made an excellent recovery. I might follow this case with another almost similar in history, in which nitro-glycerine was used with as good results; but in a third case it did no good. Taking it all in all, however, my experience is that nitro-glycerine is one of the best remedies we can use in these circumstances.

Dr. Connell: Two such cases came under my notice. One showed about ten per cent. albumen; this was at the beginning of the seventh month. In about two weeks I was called to see her, in convulsions. Another case was one in which I was called by Dr. Hallock. In this case there was no albumen in the urine. The woman had reached full term and the labor progressed slowly but favorably; being first labor, of course it was a little tedious. During the second stage she was seized with terrific convulsions; during the time we were delivering her, which we did as quickly as possible, she had three or four convulsions. There was no trace of albumen in her urine.

Dr. Stewart: Albuminuria, uræmia and convulsions during pregnancy are invariably ascribed to circulation interferences by pressure of the enlarged uterus. But it is worthy of note that abdominal tumors as large as the uterus at term, and occupying the same situation, do not entail these results. How can this be explained? It is to be remembered that pregnancy possesses other means to effect convulsions, namely, through the nervous system. In addition, discrimination is necessary, and all convulsions occurring during pregnancy, labor, and the lying-in, are by no means to be ascribed to, and treated as, the result of uræmia.

Dr. Green: April 28th last, I was summoned to see a child six years of age, with typhoid fever. The patient remained ill with the characteristic temperature curves during twenty days. During the last six days, cerebral symptoms seemed to predominate, and about the time the fever subsided and the temperature became normal, the child seemed to convalesce rapidly, and seemed cheerful. On the twenty-second day, when I called they told me the child had not spoken the past night, and had spoken but once since I made my visit the day before. She continued in this mute condition for six days without uttering an audible note. During this time there did not seem to be any unusual delirium, but about twenty-four hours before she began to speak she manifested considerable delirium of rather a cheerful nature. It was on the seventh day she began again to speak, and within forty-eight hours she talked as usual. I think this case is unusual. The child made a good recovery, and is now apparently as well as before the illness.

Dr. Buchanan: I have seen a case very similar to Dr. Green's. A child seven years old was attacked by typhoid fever, and passed through a typical course of fever lasting four weeks. The child was then unable to speak. It had no other cerebral symptoms whatever. In all other respects the case was an ordinary one. It returned to speech more gradually than Dr Green's case, but finally completely recovered. I think it was almost a week from the time it commenced to say individual words until it was able to express its wants.

Dr. Huselton: I have to report a case of dislocation of the hip joint. The dislocation was that which is commonly known as the dorsal dislocation. The case had been seen and manipulated by another surgeon, and in the manipulation the head of the bone had slipped into the thyroid foramen, an accident which may happen to any one of us, and which is not so readily reduced. On my third effort, I succeeded in dislodging the bone, and it returned to its place with a snap so loud that I felt certain I had fractured the neck, but was glad to find that I had not. This is the third case of dislocation of the hip I have reduced by manipulation. The first one I had no difficuly with whatever. The second one I also succeeded in reducing without special effort. The patient made a quick recovery in this last case.

Dr. McCann: This is not an uncommon accident in attempting to reduce a dislocation of the hip joint; in the effort to place the head of the bone in its normal position, unless there be extreme care exercised, it will be thrown into one of the other dislocations. Some years ago a man was struck on the back by a railway train, and sustained a dislocation of the hip, as well as other injuries. When he was brought into the hospital it was not appreciated that he was fatally injured, and an effort was made to reduce the dislocation. The effort most signally failed for a long time; finally the bone slipped into position just as the man was dying.

A post-mortem showed that not alone the fibro-cartilage around the periphery of the acetabulum, but a bony section at the superior edge also, was fractured off. The specimen was not retained. It would have been of value. A few years ago a child of ten years was subjected to several efforts at reduction of the dislocation by manipulation. The head of the bone could be thrown into the thyroid foramen, and was apparently reduced, but as soon as the extension was removed there was an immediate reproduction of the dislocation, although a deformity did not exist. After two or three surgeons had made a number of efforts to reduce the dislocation by manipulation, the child was put in bed with extension by a splint, and eventually recovered. I am satisfied that this case—although, fortunately for the child, we had no opportunity to verify the opinion, for she lived—was also a case in which the cartilage, and perhaps some bone, had been torn off.

Occasionally, as has been demonstrated by Dr. Murdoch, dislocation which cannot be reduced by the ordinary manipulations, will be reduced by a little traction. I remember one in which I was able, by my own unaided effort, catching the foot and pulling powerfully upon the limb, to replace the head of the bone in the socket.

Dr. Pettit: Two cases I saw in the hospital which were reduced by extension. One had been worked with a long time by a half-dozen physicians and attendants, and there was failure to reduce the dislocation. While they were rigging up the rope and pulley arrangement to try, some one grasped the man by the limb, and by no great force, but simply by steady pulling for maybe three-fourths of a minute, the bone slipped into place. Since that time I have seen one other case reduced in the same manner, after quite a good deal of manipulation without success, by extension without a great deal of force, but the force being kept up some minutes steadily.

Dr. Buchanan: I recall a case in which efforts were made to reduce a dislocation of the hip in a railroad man, by, I suppose, five or six competent surgeons, and when they were through, the reduction was unaccomplished, and the opinion was expressed that it must be a fracture of the rim of the acetabulum neck, and that it was impossible to reduce it. Dr. LeMoyne requested to be permitted to put on an extension, and by extension and some little manipulation, not the ordinary manipulation, but manipulation during extension, he reduced this hip joint. This man was on the point of being returned to bed with his luxation unreduced when the successful attempt was made.

Dr. Batten: I had a case of dislocation of the hip, and could not reduce it. Dr. Emmerling, Dr. Dickson and Dr. Reiter were present. The bone went into place with a snap under Dr. Reiter's manipulation.

Dr. Kœnig: I would like to ask if it might not be possible that the position of the rent in the capsule has much to do with the return of the bone; where the surgeon is not able to return the bone by the method of manipulation, and where the method of extension is readily followed by reduction of the bone, is not the location of the rent a factor in the case?

Dr. McCann: What I wish to say is, that in the cases of extension, the patient being entirely under the influence of an anæsthetic, completely relaxed, the amount of force used was not great. In my own cases I did not exert a very great force, and it slipped in.

Another method consists in etherizing the patient, laying him on his abdomen on a table, and allowing the limb to hang down over the end of the table. The result is that after a certain length of time the muscles of the abdomen relax, and with almost no manipulation the head of the bone is thrown into its proper position.

Dr. Huselton: A farmer sustained a simple dislocation of the shoulder. He was taken into the town of Harmony, where two surgeons tried to reduce the dislocation. They failed, and had the man loaded into a spring wagon and sent to my office, with a note asking me to call in some of my friends and attempt to reduce the dislocation, and in the event of a failure, to send him to the West Penn Hospital. I said I would make an attempt to reduce it myself. I took the patient into my back office, laid him upon the lounge, and standing behind him, I pulled and told him to pull, which he did, and I think the dislocation was reduced in about two minutes. Now these were competent men who failed in this case, and they seemed to have exhausted every effort. Therefore I think we should be very charitable indeed before we condemn a physician for failure to reduce a dislocation.

THREE CASES OF FATAL PERICARDITIS, WITH AUTOPSIES IN TWO.

Dr. Lange: I wish to report three fatal cases of pericarditis. The first was a large German, previously healthy, with no discoverable hereditary taint, except that his mother had died of some lung disease at the age of thirty-two. This man was a a cooper by occupation and boasted of his previous excellent health. He presented, on examination, the usual signs of pericarditis, except that of pain. He presented, in addition, the ordinary signs and symptoms of a slight fever. Pericarditis was suspected in this case, and during the next three weeks under observation it was ascertained to exist. This man died of heart failure in the water closet. The autopsy showed the heart to be the so-called hairy heart. There were numerous and strong adhesions between the parietal and visceral layers. Other lesions, consisting of organized bands from the visceral to the pareital pericardium of from a quarter to an inch in length, and as strong as the chordæ tendineæ. These must have exerted a potent and pernicious influence upon the contraction and rotation of the organ. Still other bands were free at one extremity which floated in the very limited amount of effusion. This was a tubercular pericarditis, and nowhere else throughout the body was tubercle discovered.

The second case occurred in an Italian aged 35, who had a left-sided, lower-lobed croupous pneumonia, accompanied by a pleuritic effusion large enough to require tapping. In the third week, resolution not happening, the pneumonia became purulent, and thus developed the secondary pericarditis. On autopsy the heart was found to be like the first, and the effusion to be small. This patient died while sitting on the edge of his bed.

The third case was an Irishman, a very active man, yardmaster at one of the railroads here. He came to my office complaining of shortness of breath and presented the signs, symptoms and phenomena of a mild fever. He had no pain, little headache, little backache, general malaise, anorexia, in short all the signs and symptoms of a slght fever, a pericardiac friction murmur, and a tumbling heart, cut no enlarged area of cardiac dullness. Dyspnœa was his only complaint. He had it two months and it was growing. After two weeks he thought himself so well that we could not restrain him. He would leave his bed and go about his room. One Sunday morning he sent for a barber to shave him. He got up, sat on a chair, took the Sunday paper to look it over and fell dead. There was no post mortem. From repeated physical examinations I believe the conditions would have been found as in other cases. In pericarditis of gravity, which cases constitute the small minority, there is one remedy to which we at last arrive. This is digitalis. It is said that digitalis is proper to strengthen the action of the heart. My experience with these cases and others leads me to think that digitalis has very little effect in increasing the power of the heart in pericarditis.

Another point is that death from pericarditis, which is usually ascribed to the size of the effusion when it is large, may be due rather to interference with the action of the heart by adhesion. It is my opinion that this latter is frequently, and that large effusions are rarely, the cause of death. Large effusions belong rather to those inflammations of the pericardium which complicate rheumatism and nephritis, and which are not fatal. When the effusion is large, what can we expect from digitalis? The interference in this case is not with contraction—but with the filling—the diastole of the heart; and this being purely a muscular relaxation is uninfluenced by digitalis. The only effect then that we should expect is that which would happen after the administration of digitalis, in for instance, the granular degeneration of typhoid fever, pneumonia, or any disease of gravity and duration; and, this, it must be confessed, is little. The effect, indeed, would be less, for even if the systole should be improved by digitalis, the diastole cannot be, and consequently the intra-arterial tension is not increased. On the other hand, if the adhesions constitute the obstacle to efficient contractions, we can understand how an increase of power in systoxle by digitalis might fracture, tear off, or free the parietal from the visceral pericardium, and thus allow an increased quantity of blood in the arterial system. But I have not observed this to happen, and the administration of digitalis for pericarditis is pregnant with disappointment, and is in striking contrast with its effects in heart dilatation.

